# A preliminary needs assessment of American Indians who inject drugs in northeastern Montana

**DOI:** 10.1186/s12954-017-0146-1

**Published:** 2017-05-08

**Authors:** Mike Anastario, Kris FourStar, Adriann Ricker, Rebecca Dick, Monica C. Skewes, Elizabeth Rink

**Affiliations:** 1 Department of Mathematics, Universidad Centroamericana José Simeón Cañas, Antiguo Cuscatlán, El Salvador; 2 Fort Peck Tribal Health Department, Poplar, MT USA; 3 Fort Peck Health Promotion Disease Prevention Wellness Program, Poplar, MT USA; 4 Children’s Hospital of Pittsburgh of University of Pittsburgh Medical Center, Pittsburgh, PA USA; 5 Department of Psychology, Montana State University, Bozeman, MT USA; 6 Department of Health and Human Development, Montana State University, 318 Herrick Hall, Bozeman, 59715 MT USA

**Keywords:** American Indians, Injection drug use, Fort Peck, HIV risk, Hepatitis C risk

## Abstract

**Background:**

Injection drug use has not been well documented in American Indians living in the USA. American Indian and Alaskan Natives (AI/ANs) show higher rates of substance use compared to the general population, and have historically been subject to a number of risk factors that are known to increase the likelihood of substance use. AI/ANs also experience increased risk for infectious diseases that are transmitted via injection drug use and/or sexual activity. Harm reduction approaches have been shown to be effective for decreasing risk of disease transmission in at-risk populations, and may be well suited for AI/AN injection drug users residing in rural reservation communities. In this study, we aimed to examine the characteristics of American Indians (AI) who use injection drugs (PWUID) in northeastern Montana to identify needs that could be addressed with harm reduction programming.

**Methods:**

For the present study, we used a respondent-driven sampling approach to generate a sample of 51 self-identified male and female injection drug users ≥18 years of age who were American Indians living on the Fort Peck Indian Reservation. Sampling weights were applied to all analyses using Respondent-Driven Sampling Analysis Tool (RDSAT).

**Results:**

There were no strong recruitment patterns by age, sex, or ethnic identity status of the recruiter or participant, but there were strong within-group recruitment patterns by location within the reservation. The majority of the sample reported initiating substance use before the age of 18. Participants reported significant risk for HIV, hepatitis, and other infectious diseases through their drug use and/or risky sexual behavior. Sixty-five percent reported having reused syringes, and 53% reported drawing from the same filter. Seventy-five percent reported inconsistent condom use during the 3 months preceding the survey, and 53% reported injecting drugs during sex during the 3 months preceding the survey. Only 66% of participants reported having been tested for HIV in the 12 months preceding the survey. The vast majority (98%) of respondents expressed interest in a harm reduction program. Seventy-six percent reported that it was easy or very easy to obtain new syringes.

**Conclusions:**

We documented several risks for blood-borne pathogens, including elevated levels of syringe reuse. Further, we documented significant interest in harm reduction interventions in the present sample of AI/AN injection drug users. Findings suggest a need for increased access to harm reduction programming for AI/AN injection drug users to reduce the transmission of infectious disease and increase access to compassionate care.

## Background

American Indian and Alaska Native (AI/AN) people in the USA experience health disparities in rates of substance use disorders and rates of some infectious diseases due to blood-borne pathogens, such as hepatitis C [1]. These disparities can often be more pronounced in tribal communities located in rural areas with limited access to services and interventions. Harm reduction interventions such as needle exchange programs have been shown to effectively reduce transmission of infectious disease and also increase access to substance abuse treatment among injection drug users [2–4]. However, these interventions remain controversial despite overwhelming evidence for their beneficial public health effects. This research involved assessing needs and interest in harm reduction programs among AI/AN injection drug users residing in a rural reservation community.

Injection drug use has not been well documented among AI/AN communities in the USA. AI/ANs show higher rates of substance use disorders and have historically been subject to structural violence—a social phenomenon that results in detriments to the bodies of selves and others [5]. Elevated rates of illicit drug use have been documented in AI/ANs, and AI/ANs experience the highest rate of drug-induced deaths among all racial/ethnic groups [6]. In particular, age-adjusted mortality rates for alcohol are elevated for AI/ANs compared to non-AI/ANs in the USA, and alcohol use is considered to be the largest contributing factor to the elevation in the premature death rate that is observed [7]. Further, non-AI/AN drinkers have been reported as drinking more frequently but in smaller quantities than AI/AN reservation samples, who were reported as engaging more often in heavy periodic drinking [7]. Adolescent substance use and methamphetamine use in particular have been documented as particular issues affecting AI/ANs [8, 9]. At the same time, there is a notable lack of access to substance abuse treatment and other quality health care services in rural reservation communities, further promulgating risk for health problems resulting from injection drug use.

In the USA, 1.7% of the population identifies as AI/AN, either alone or in combination with other races [10]. Within this subset of the US population, injection drug use has not been well documented. However, existing data suggests that injection drug use may disproportionately affect AI/AN communities. Whites and AI/ANs have shown a higher proportion of HIV cases occurring among PWUID men who have sex with men [11]. Further, illicit drug use during the past month was higher in AI/AN youth in comparison to all other single ethnic/racial identity classifications [12]. In another study that used multiple datasets from programs targeting individuals at high risk for substance abuse, AI/ANs in Los Angeles county exhibited younger ages of onset of alcohol, marijuana, methamphetamine, and “other” drug use, and an elevated mean number of recent illicit drug injections [13]. In one community-based mail survey of 155 urban American Indian women, 6% reported ever having injected nonprescription drugs and 19% reported sex with an injection drug user [14]. Further, risk factors for substance abuse that reflect patterns of structural violence against indigenous people, such as poverty, unemployment, exposure to violence, early childhood adversity, and trauma [15–19], are likely to contribute to greater rates of injection drug use within AI/AN communities. These findings illustrate the need for further research regarding injection drug use in AI/AN communities.

In this study, we aimed to examine risk factors for infection by blood-borne pathogens among native people who use injection drugs (PWUID) in northeastern Montana and to examine needs that may be addressed with harm reduction programming. The Fort Peck Tribal Council asked the research team to complete the study as a result of the elevated hepatitis C rate on the reservation. AI/ANs are not a culturally homogenous group, as there are 566 federally recognized tribes living in the USA [20]. The Fort Peck Reservation in northeastern Montana is home to approximately 8000 Nakoda (Assiniboine) and Dakota & Lakota (Sioux) nation members. In this study, we conducted a preliminary survey of American Indian (AI) PWUIDs on the Fort Peck Reservation, documenting recruitment patterns, demographics, substance use behaviors, and other psychosocial risk factors that could lead to infection with blood-borne pathogens. Results will have implications for future studies and health programming on the Fort Peck Reservation.

## Methods

### Population and sampling

The present study took place on the Fort Peck Reservation in northeastern Montana. The reservation comprises 2.1 million acres. The Fort Peck Reservation is characterized as a young population with 44% of the inhabitants under the age of 25. Low educational attainment, high unemployment, poverty, and social problems including crime and drug use also are present on the Fort Peck Reservation [21, 22].

For the present study, the population of interest was self-identified male and female injection drug users ≥18 years of age who were members of the Assiniboine and Sioux Tribes. We used a chain-referral sampling approach to generate our sample. The chain-referral approach was selected on the assumption that members of the target population know one another and are densely interconnected [23, 24]. The chain-referral sampling approach began with one male seed participant. Participants were recruited through word of mouth and invitation by the previous participant. The recruitment criteria included injecting drugs within the past 3 months and being a Fort Peck tribal member. A screener was not used prior to questionnaire administration. Participants consented to complete a self-report questionnaire and received a $20 cash incentive for participation, which took approximately 1 h. Participants were provided two recruitment cards each and were asked to recruit other PWUIDs, for which they would receive an additional $10 cash incentive. The final sample yielded 51 PWUIDs.

Institutional Review Broad approval for the study was received from Montana State University. At the time this study was initiated, the Fort Peck Tribal Council served as the Fort Peck Tribes’ Institutional Review Board and also gave approval for the study. Since the completion of the study, Fort Peck established a formal Institutional Review Board, which is administered through Fort Peck Community College. The tribal IRB reviewed and approved this manuscript prior to submission.

### Measures

Participants were administered a face-to-face questionnaire by one male interviewer. The questionnaire was developed by project staff using established measures for demographics, drug and alcohol use, social networking, harm reduction program opinions, knowledge of hepatitis C and HIV, psychosocial factors, and sexual practices.

Demographic questions included respondent sex, age, highest level of education completed, and municipal location of the respondent on the reservation. In order to protect the identity of participants, municipal location names have been masked in the results and tables of this manuscript.

Questions concerning drug and alcohol use included history of drug use, frequency of injection drug use over the previous 6 months, practices of reusing equipment/works, level of ease in obtaining new syringes, frequency with which respondents obtain new syringes, measures taken to clean syringes, and frequency with which respondents cleaned spoons or obtained new spoons.

Social networking questions included the social role of the person/people with whom respondents inject drugs (e.g., their relationship to the respondent), whether money is pooled together by people in the drug user’s social network to obtain drugs, and suggested mechanisms for getting health promotion information out to other injection drug users.

Harm reduction needs assessment questions included interest in a potential harm reduction program (yes/no), suggested mechanisms for setting up a harm reduction program (where respondents were given a choice of the following responses: office setting, outreach (van), outreach (calling in office and delivery), and peer models), and types of preferred services to be received from a potential harm reduction program (where respondents were given a choice of the following responses: information regarding safely injecting, free testing for sexually transmitted infections (STIs)/hep C/HIV, referrals to counseling centers, information regarding STIs, and support groups).

HIV and hepatitis C knowledge questions were adapted from other health questionnaires and included a series of 24 true/false questions regarding hepatitis C, and 17 true/false questions regarding HIV. For all knowledge items, respondents had the opportunity to select “Don’t know.” The percent of respondents with the correct answer for a given knowledge item was the variable analyzed. Given that all items were dichotomous, we used the Kuder Richardson-20 statistic [25] to examine the internal consistency of items within each knowledge domain (0.77 for hepatitis C, 0.66 for HIV).

Psychosocial factors included perceived level of risk of getting HIV from having sex with a sex partner without using a condom, level of importance in using condoms, intention to use condoms when having sex to avoid contracting HIV, likelihood of using a condom in the next 3 months to prevent getting HIV and other sexually transmitted diseases (two separate questions), frequency of communication with partners regarding condoms, and whether there is worry over contracting hepatitis C and HIV. Psychosocial factors were measured using a perception based Likert Scale (not at all likely, a little likely, moderately likely, very likely, extremely likely), and responses were dichotomized for those who felt ≥moderately likely (1), or less than moderately likely (0) regarding a given item. Each item was analyzed separately.

Sexual behavior included the number of sexual partners over the participant’s lifetime (dichotomized at >10 given the average number of sexual partners estimated for Generation X individuals [26]), the number of sexual partners over the past 3 months (dichotomized at ≥2 sexual partners to approximate multiple sexual partnerships within this period), evidence of injection drug use during sex over the past 3 months, and inconsistent condom use. Individuals were also asked whether they had been tested for an STI or HIV in the 12 months preceding the survey.

### Data analysis

Weights were applied to all univariate analyses using the Respondent-Driven Sampling Analysis Tool (RDSAT) [27], which assists with adjustment for recruitment bias in peer-referral sampling [28]. As this was a preliminary study, we first examined recruitment patterns by examining transition probabilities for select demographic factors. To generate estimates of population proportions, bootstrap confidence intervals for univariate analyses were derived from 15,000 re-samplings per variable. The average network size of PWUIDs in this sample was set to 10 as personal network size was not collected during fielding of the chain-referral sample. All estimates were reported as percentages. Bivariate relationships were explored using correlations and odds ratios.

## Results

### Demographic characteristics

Table 1 illustrates demographic characteristics of recruits relative to the person who recruited them for participation. There were no strong within-group recruitment patterns by age or sex of the recruiter/recruit (Table 1). As expected, there were strong within-group recruitment patterns by location within the reservation, particularly for recruiters located in three of the municipalities examined (Table 1).

**Table 1:** Characteristics of American Indians who use injection drugs in northeastern Montana by characteristics of recruiter, *n* = 51

Characteristics						
	Age of recruit					
Age of recruiter	18–24	25–30	31–36	37–42	43+	TOTAL
18–24	.33	.39	.06	.17	.06	1
25–30	.31	.15	.15	.15	.23	1
31–36	.50	0	0	.33	.17	1
37–42	.25	.50	.13	.13	0	1
43+	.25	.25	.25	.25	0	1
Total distribution	.33	.29	.10	.18	.10	1
	(16)	(14)	(5)	(9)	(5)	(49)
Equilibrium	.32	.29	.11	.18	.10	1
	Sex of recruit					
Sex of recruiter	Female	Male				TOTAL
Female	.50	.50				1
Male	.55	.45				1
Total distribution	.53	.47				1
	(26)	(23)				(49)
Equilibrium	.53	.48				1
	Location of recruit					
Location of recruiter	Munic. 1	Munic. 2	Munic. 3	Munic. 4	Other	TOTAL
Municipality 1	.20	.20	.20	.20	.20	1
Municipality 2	.11	.78	.11	0	0	1
Municipality 3	0	.08	.84	.05	.03	1
Municipality 4	0	0	.33	.67	0	1
Other	.20	.20	.20	.20	.20	1
Total distribution	.02	.20	.67	.08	.02	1
	(1)	(10)	(33)	(4)	(1)	(49)
Equilibrium	.04	.26	.54	.13	.03	1

Thirty-two percent of respondents were 18–24 years of age, 29% were 25–30 years of age, 11% were 31–36 years of age, 18% were 37–42 years of age, and 10% were ≥43 years (Table 2). Forty-eight percent of the sample was male, and 32% had completed high school. The majority of the sample lived in municipality #1 (54%), followed by municipality #2 (26%), municipality #3 (13%), municipality #4 (4.3%), and other locations (3%) (Table 2).

**Table 2:** Demographic characteristics of American Indians who use injection drugs in northeastern Montana, *n* = 51

Characteristics	Estimated population proportion (95% CI)
Age	
18–24	.32 (.19–.45)
25–30	.29 (.19–.39)
31–36	.11 (.04–.19)
37–42	.18 (.08–.29)
43+	.10 (.04–.18)
Sex	
Male	.48 (.35–.61)
Female	.53 (.39–.65)
Completed high school	.32 (.18–.47)
Lives in:	
Brockton	.043 (.0–.14)
Poplar	.26 (.0–.65)
Wolf Point	.54 (.1–.84)
Frazer	.13 (.0–.41)
Other	.03 (.0–.11)

*CI* confidence interval

### Drug use

All participants reported ever having used needles to inject drugs (Table 3). In the 6 months preceding the survey, 86% reported injecting drugs once in a while, occasionally, or regularly. Nine percent were unsure of the frequency with which they injected, and 5% reported not injecting drugs in the month preceding the survey. Sixty-five percent reported ever having reused syringes, and 53% reported drawing from the same filter. Twenty-four percent of respondents reported that it was difficult to obtain new syringes, and 43% reported obtaining new syringes less than once a week. Thirty-five percent reporting doing nothing at all to clean syringes, 20% reported rinsing the used syringe with water, 32% rinsing with bleach, and 44% reporting using an alcohol wipe on the syringe. Five percent reported never using a new or cleaned spoon. The majority reported initiating substance use before the age of 18 for general drug use (67%) and alcohol use (82%). The plurality of the sample began injecting drugs between 18 and 24 years of age (47%) (Table 3).

**Table 3:** Drug and alcohol use in American Indians who use injection drugs in northeastern Montana, *n* = 51

Characteristics	Estimated population proportion (95% CI)
Has used needles to inject drugs	1.0 (1.0–1.0)
In the past 6 months, frequency with which injected drugs	
None	.05 (.0–.13)
Once in a while (<1/week)	.38 (.22–.54)
Occasional (1–2/week)	.28 (.18–.39)
Regular (3 or more times in a week)	.20 (.09–.32)
Unsure	.09 (.02–.17)
Has reused syringes	.65 (.48–.82)
Draws from the same filter (sponge/cotton)	.53 (.40–.65)
Level of ease in obtaining new syringes	
Very easy	.39 (.25–.53)
Easy	.37 (.27–.47)
Difficult	.24 (.12–.37)
Very difficult	0
Frequency with which obtains new syringes	
Never	.05 (.0–.13)
Once in a while (<1/week)	.38 (.22–.55)
Occasional (1–2/week)	.28 (.18–.39)
Regular (3 or more times in a week)	.20 (.08–.32)
Only on the weekends	.09 (.02–.17)
Unsure	0
Measures used to clean syringes	
Rinse the used syringe with water	.20 (.1–.33)
Rinse with bleach	.32 (.17–.47)
Alcohol wipe	.44 (.28–.61)
Nothing at all	.35 (.12–.61)
Frequency with which cleans spoon, or uses a new spoon	
Never	.05 (.0–.14)
Once in a while (<1/week)	.12 (.02–.25)
Occasional (1–2/week)	.10 (.02–.20)
Regular (3 or more times in a week)	.67 (.52–.80)
Unsure	.06 (.0–.13)
Age at which first started using drugs	
<18	.67 (.55–.78)
18–24	.21 (.12–.31)
>24	.12 (.04–.22)
Age at which first started drinking	
<18	.82 (.69–.92)
18–24	.19 (.08–.31)
>24	0
Age at which first started using injection drugs	
<18	.16 (.08–.25)
18–24	.47 (.30–.63)
>24	.37 (.22–.53)

*CI* confidence interval

### Social networking and harm reduction program opinions

Respondents most frequently reported injecting with their friends (55%), followed by their sex partners (49%), family (35%), or anybody at all (2%) (Table 4). Thirty-one percent reported injecting alone. Seventy-seven percent reported pooling money to obtain drugs. Nearly all participants (98%) reported that they would use a harm reduction program. The preferred way to get information out to injection drug users was by word of mouth (92%). Ninety-four percent said that they would prefer a peer model for the delivery of a potential harm reduction program. All participants said that as part of a harm reduction program they would like to see free testing for STIs, hepatitis C, and HIV along with information regarding STIs (Table 4). Three participants specifically stated that they would like to see Suboxone made available through a harm reduction program.

**Table 4:** Social networking and harm reduction program opinions in American Indians who use injection drugs in northeastern Montana, *n* = 51

Characteristics	Estimated population proportion (95% CI)
Usually injects with	
Family	.35 (.19–.51)
Sex partner	.49 (.34–.63)
Friends/others	.55 (.43–.68)
Anybody at all	.02 (.0–.06)
Self	.31 (.20–.42)
Pools money together to obtain drugs	.77 (.57–.92)
Best way to get information out to IV drug users	
Newspaper	.72 (.57–.86)
Word of mouth	.92 (.84–.98)
Radio	.38 (.22–.53)
Posters around the community	.57 (.37–.76)
Would utilize a harm reduction program	.98 (.94–1.0)
Preference for a potential harm reduction program	
Office setting	.47 (.31–.63)
Outreach (van)	.81 (.63–.94)
Outreach (calling in to the office and delivery)	.87 (.71–.98)
Peer models	.94 (.88–1.0)
Types of services would like to see at a harm reduction program	
Information regarding safely injecting	.92 (.82–1.0)
Free testing for STIs/Hep C/HIV	1.0
Referrals to counseling centers	.82 (.71–.92)
Information regarding STIs	1.0
Support groups	.92 (.85–.98)

*CI* confidence interval

### Knowledge of hepatitis C and HIV

Concerning knowledge of hepatitis C, participants were generally knowledgeable that hepatitis could be spread through sharing injecting equipment (85% correct), blood-to-blood contact (90% correct), and that a person infected with hepatitis C may not have any symptoms of the disease (88% correct) (Table 5). There was lower knowledge that hepatitis C was not caused by bacteria (35% correct), that sexual transmission was not a common way that hepatitis C is spread (23% correct), that most people infected with hepatitis C will not die prematurely because of infection (28% correct), and that there is a vaccine for hepatitis C (42% correct) (Table 5). The unweighted sample mean for the average number of correct hepatitis C answers was 59%. Hepatitis C knowledge showed some associations with behaviors. Overall knowledge of hepatitis C was positively correlated with the reported level of ease in obtaining new syringes (rho = .23, *p* = 0.099). Further, the odds of inconsistent condom use increased with increasing overall levels of hepatitis C knowledge (OR = 13.0, 95% confidence interval (CI) .34–502.0, *p* = 0.168).

**Table 5:** Knowledge of hepatitis C in American Indians who use injection drugs in northeastern Montana, *n* = 51

Characteristics	Estimated population proportion that correctly answered item (95% CI)
Sexual transmission is a common way hepatitis C is spread.	.23 (.10–.36)
Most people who get hepatitis C will die prematurely because of infection.	.28 (.15–.42)
Hepatitis C is a mutation of hepatitis B.	.30 (.17–.44)
Hepatitis C is now one of the leading reasons for liver transplantation in Australia.	.32 (.18–.47)
Hepatitis C is caused by a bacteria.	.35 (.22–.48)
People with hepatitis C should be restricted from working in the food industry.	.37 (.22–.51)
There is a vaccine for hepatitis C.	.42 (.27–.59)
Once you have had hepatitis C you cannot catch it again because you are immune.	.52 (.39–.65)
HIV is easier to catch than hepatitis C.	.52 (.41–.63)
Having a medical and/or dental procedure performed in the Middle East, Southeast Asia or the Mediterranean increases a person’s chances of contracting hepatitis C.	.52 (.42–.61)
There is a pharmaceutical treatment available for hepatitis C.	.53 (.40–.67)
Hepatitis C can be spread by mosquitoes.	.56 (.43–.68)
An individual can have hepatitis C antibodies without being currently infected with the virus.	.58 (.47–.71)
Hepatitis C is caused by a virus.	.69 (.55–.84)
Hepatitis C is associated with an increased risk of liver cancer.	.70 (.56–.82)
People with hepatitis C should restrict their alcohol intake.	.74 (.58–.88)
Hepatitis C can lead to cirrhosis	.75 (.65–.86)
Some people with hepatitis C were infected through blood transfusions.	.76 (.61–.88)
Some people with hepatitis C were infected through unsterile tattooing.	.78 (.57–.94)
Hepatitis C can be spread through close personal contact such as kissing.	.78 (.69–.88)
Hepatitis C can be spread through sharing injecting equipment, such as needles, tourniquets, spoons, filters and swabs.	.85 (.57–1.0)
Hepatitis C is spread through the air in enclosed environments like crowded uses and elevators.	.86 (.78–.94)
A person can be infected with hepatitis C and not have any symptoms of the disease.	.88 (.78–.96)
Hepatitis C is spread through blood-to-blood contact.	.90 (.76–.98)

*CI* confidence interval

For knowledge of HIV (Table 6), participants were generally knowledgeable that pulling out the penis before a climax is not a preventive measure (90% correct), that there is not a vaccine (86% correct), that antibiotics are not protective against infection with HIV (89% correct), and that using Vaseline or baby oil with condoms does not lower the chance of getting HIV (80% correct). There was lower knowledge regarding that all pregnant women infected with HIV would not have babies born with AIDS (23% correct) and lower knowledge that there is a female condom that can help decrease a woman’s chance of getting HIV (45% correct) (Table 6). Women and men were equally knowledgeable about use of the female condom (results not reported in table). The unweighted sample mean for the average number of correct HIV answers was 71.2%. HIV knowledge showed some associations with behaviors. Overall knowledge of HIV was positively correlated with the reported level of ease in obtaining new syringes (rho = .25, *p* = 0.071). The odds of inconsistent condom use increased with increasing overall levels of HIV/AIDS knowledge (OR = 25.8, 95% CI .53–1263.2, *p* = 0.101).

**Table 6:** Knowledge of HIV in American Indians who use injection drugs in northeastern Montana, *n* = 51

Characteristics	Estimated population proportion that correctly answered item (95% CI)
All pregnant women infected with HIV will have babies born with AIDS.	.23 (.10–.38)
There is a female condom that can help decrease a woman’s chance of getting HIV.	.45 (.29–.61)
Coughing and sneezing *do not* spread HIV.	.53 (.40–.67)
People are likely to get HIV by deep kissing, putting their tongue in their partner’s mouth, if their partner has HIV.	.59 (.44–.74)
Taking a test for HIV 1 week after having sex will tell a person if she or he has HIV.	.60 (.47–.72)
People who have been infected with HIV quickly show serious signs of being infected.	.65 (.51–.78)
A natural skin condom works better against HIV than does a latex condom.	.68 (.53–.80)
A person can get HIV from oral sex.	.71 (.58–.83)
A person can get HIV by sitting in a hot tub or a swimming pool with a person who has HIV.	.72 (.57–.84)
b. A person can get HIV by sharing a glass of water with someone who has HIV	.76 (.65–.86)
Showering or washing one’s genitals/private parts, after sex keeps a person from getting HIV	.77 (.51–.96)
Using Vaseline or baby oil with condoms lowers the chance of getting HIV.	.80 (.66–.92)
A woman cannot get HIV if she has sex during her period.	.81 (.73–.90)
A woman can get HIV if she has anal sex with a man.	.83 (.69–.94)
There is a vaccine that can stop adults from getting HIV.	.86 (.78–.94)
A person will *not* get HIV if she or he is taking antibiotics.	.89 (.71–1.0)
Pulling out the penis before a man climaxes/cums keeps a woman from getting HIV during sex.	.90 (.82–.98)

*CI* confidence interval

### Psychosocial factors

The majority of participants (≥79%) perceived that there were ≥moderately likely consequences of becoming infected with HIV and STDs when not using a condom (Table 7). However, only 54% reported that they were at least moderately likely to use a condom when having sex to prevent HIV, and 48% reported being at least moderately likely to use a condom to prevent an STI other than HIV. Forty-nine percent reported communicating with a partner with at least moderate frequency about using condoms. Eighty-five percent were worried about contracting hepatitis C or HIV by injecting drugs (85%) (Table 7).

**Table 7:** Psychosocial factors in American Indians who use injection drugs in northeastern Montana, *n* = 51

Characteristics	Estimated population proportion (95% CI)
≥Moderately likely could get HIV from having sex with a sex partner without using a condom	.79 (.67–.9)
≥Moderately likely could get a STD other than HIV from having sex with a sex partner without using a condom	.82 (.69–.94)
≥Moderately important to use condoms when having sex with a sex partner	.80 (.65–.92)
≥Moderately likely that in the next 3 months will use a condom when having sex to prevent getting HIV	.54 (.38–.70)
≥Moderately likely that in the next 3 months will use a condom when having sex to prevent getting a sexually transmitted disease other than HIV	.48 (.31–.64)
≥Moderately frequency with which communicates with a sex partner about using a condom	.49 (.35–.63)
Worried about contract hep C/HIV by injecting	.85 (.76–.92)

*CI* confidence interval

### Sexual practices

As it regards decision making about whether or not to use a condom during sex, 51% reported that they alone were the person who makes the decision, 21% reported that their partner makes the decision, and 28% reported that they make the decision with their partner (Table 8). Men were more likely to report making the decision to use condoms *alone* in comparison to women (results not reported in table). Eighteen percent of participants reported ≥2 sexual partners during the 3 months preceding the survey. Seventy-five percent reported inconsistent condom use during the 3 months preceding the survey, and 53% reported injecting drugs during sex during the 3 months preceding the survey. Only 66% of participants reported having been tested for HIV in the 12 months preceding the survey. In total, 59% reported having sex with a partner who knew or was suspected of having sex with another person, shared needles to shoot drugs, had an STD, or had HIV/AIDS in the 12 months preceding the survey (Table 8).

**Table 8:** Sexual practices of American Indians who use injection drugs in northeastern Montana, *n* = 51

Characteristics	Estimated population proportion (95% CI)
Who makes decision about whether or not to use a condom	
Me	.51 (.37–.65)
My partner	.21 (.10–.31)
We both do	.28 (.16–.43)
>10 sex partners during lifetime	.57 (.46–.69)
≥2 sex partners during past 3 months	.18 (.08–.31)
Inconsistent condom use during past 3 months	.75 (.64–.86)
Injection drug use during sex over the past 3 months	.53 (.41–.66)
Has been tested for an STD in the past 12 months	.77 (.67–.86)
Has been tested for HIV in the past 12 months	.66 (.55–.76)
In the past 12 months, had sex with a partner who	
Knew or suspected was having sex with another person	.21 (.12–.30)
Knew or suspected shared needles to shoot drugs	.27 (.16–.37)
Knew or suspected had an STD	.11 (.04–.18)
Knew or suspected had HIV or AIDS	0

*CI* confidence interval

## Discussion

In this preliminary survey of native PWUIDs on the Fort Peck Reservation in northeastern Montana, within-group demographic recruitment patterns, risks for blood-borne pathogens, and needs for harm reduction programming were documented. The gaps identified include wide-scale syringe reuse, difficulty in obtaining new syringes, and high rates of sexual risk behavior that correlate with low levels of knowledge concerning HIV and hepatitis C. This current study of injection drug use among the Assiniboine and Sioux Tribes is an important contribution to harm reduction discourse considering the dearth of literature on injecting drug practices in AI/ANs.

First, this study provided evidence of risk for blood-borne pathogens among PWUID of the Assiniboine and Sioux Tribes. The majority of participants reported having reused syringes, and there was a relatively low HIV testing rate. The evidence of appreciable risk behavior coupled with a low HIV testing rate raises concern regarding the true prevalence of HIV and hepatitis C in this population. Further, a notable portion (24%) of participants still reported that it was difficult to obtain new syringes, which suggests that accessibility to existing harm reduction programming is an issue that needs to be addressed on the reservation. Syringe reuse patterns in particular have been shown to vary with the implementation of harm reduction programs made available to PWUIDs [29, 30]. Singer et al. found successive decreases in syringe reuse following the legalization of pharmacy sale of syringes without a prescription combined with the local availability of syringe exchange programs [29]. Making syringe exchange legal and available on the Fort Peck Reservation could have a similar effect.

Our study identified gaps in knowledge and testing regarding HIV and other STIs that could be addressed by an outreach program. In particular, we identified high rates of inconsistent condom use and high rates of injecting drugs with a sex partner, as well as injecting during sex. Public health workers on the reservation should consider screening for injection drug use during sex to tailor preventive messaging to clients who may be participating in harm reduction efforts. Further, we identified evidence of discord between risk perception and behavioral intentions concerning condom use in this population. Roughly 79% of participants perceived a high risk of infection with HIV and STIs when not using a condom, but a considerably smaller percentage reported that they would use a condom to prevent these diseases. In a post hoc logistic regression for inconsistent condom use, we found that the likelihood of inconsistent condom use decreased with intention to use a condom (adjusted odds ratio (aOR) = 0.44, 95% confidence interval (CI) .26–.73, *p* = 0.001) and increased with risk perception (aOR = 2.4, 95% CI 1.3–4.4, *p* = 0.006). That is, greater stated intentions to use condoms were associated with less reported inconsistency in condom use, and greater risk perception was associated with greater inconsistent condom use. It may be that participants understood the importance of using condoms for preventing disease transmission, but had poor self-efficacy that they would be capable of doing so during sexual encounters. Sexual risk reduction self-efficacy has been previously linked to lower sexual risk among injection drug users [31]. Consistent condom use has also shown a relationship with condom use self-efficacy among young males on the Fort Peck Reservation [32]. High risk perception coupled with low self-efficacy suggests that client-oriented sexual risk reduction strategies could be potentially beneficial to reduce the sexual risks evident within this PWUID population.

Based on our findings, other substances were initiated at a younger age (<18 years) in comparison to the age at which injecting drugs were first initiated. Illegal substance use has been documented as elevated among American Indians in comparison to the national average, and AI/ANs experienced the highest rates of drug-induced deaths among all racial/ethnic groups in the USA [6]. Further, early drug initiation has been documented among American Indian and Black American youth, who were more likely to initiate marijuana in comparison to white youth [33]. It is important to understand that early drug-use initiation and elevated drug-related mortality illustrate two points in the life course at which native communities may exhibit the outcomes of historically situated structural violence, which continues to occur as the traumatic ramifications of colonization continue to emerge. In this context, the inert violence of larger factors (e.g., colonization, land infringement, and the degradation of community practices and values) are conserved, or paid for, by the active violence of the people (e.g., detriments to the bodies of selves and others) [5]. Risk factors for drug use, such as poverty, violence victimization, and trauma, disproportionately affect AI/ANs [15–19]. Interventions that aim to decrease substance use in native communities should avoid taking punitive approaches that are likely to reinforce historical structural violence and that elevate harm within the community. Harm reduction frameworks offer more compassionate and effective solutions.

Finally, this study identified strong within-group recruitment patterns by municipal location among Assiniboine and Sioux Tribe members within a single reservation, particularly for recruiters located in three of the municipalities examined. These results are important for informing future efforts that use chain-referral sampling, as within-group recruitment could similarly be driven by recruiter location. We briefly consider these recruitment results in the context of community-based participatory research (CBPR), a research strategy that emphasizes the role of the community as equal partners in all phases of the research process [34]. Our research team has been employing CBPR principles for over a decade to establish a functional and respectful research partnership between members of the Assiniboine and Sioux Tribes of the Fort Peck Reservation and the university. The request for this study, and the study’s design, administration, and presentation of findings integrally involved community members and leaders. Establishing these types of community partnerships are necessary to respectfully and successfully implement and evaluate harm reduction interventions that aim to recruit and evaluate indigenous PWUID. The municipal within-group recruitment patterns likely reflect a level of trust that was built upon this collaborative research model.

### Limitations

Our study has several limitations. First, our results can only be generalized to the population of Assiniboine and Sioux Tribe members who are injection drug users on the Fort Peck Reservation in Montana. Results cannot be generalized to other AI/ANs living on other reservations. However, the results do provide key information that would inform a future study of a very hard to reach and relatively understudied population of native PWUIDs, particularly with regard to recruitment patterns and identified needs among PWUIDs. Second, the validity of the inferences in this study is limited due to our estimating network sizes at 10 and not collecting true personal network size data at the time of sampling [15]. Future studies of PWUIDs in Fort Peck should collect and track personal network sizes of each participant. Further, future assessments may benefit from collecting serological data in addition to behavioral data in order to estimate the seroprevalence of HIV and hepatitis C among PWUIDs in Fort Peck.

## Conclusions

In this preliminary needs assessment of injection drug use among Assiniboine and Sioux Tribe members on the Fort Peck Reservation in northeastern Montana, we documented elevated levels of syringe reuse and several risks for HIV and hepatitis C, which are public health concerns that could be addressed with harm reduction programming. We also identified specific gaps in knowledge and psychosocial determinants of risk behavior, which suggest the need for a client-centered approach to prevention. High within-group recruitment patterns occurred by location and will likely resurface in future studies that use chain-referral sampling. The majority of respondents expressed interest in a harm reduction program. We conclude that a harm reduction program including needle exchange, HIV testing, condom distribution, substance abuse counseling, and medication-assisted treatment for drug dependence is needed on the reservation and would be utilized by injection drug users. This type of program may go far toward addressing the public health problems associated with injection drug use and may result in decreased transmission of infectious disease as well as increased access to substance abuse treatment and other health care services. Future research is needed to examine barriers to implementing harm reduction interventions on the reservation.

### Ethics, consent, and permissions

All subjects were asked to provide their written consent prior to participating in this study. Institutional Review Broad approval for the study was received from Montana State University. At the time this study was initiated, the Fort Peck Tribal Council served as the Fort Peck Tribes’ Institutional Review Board and also gave approval for the study. Since the completion of the study, Fort Peck established a formal Institutional Review Board, administered through Fort Peck Community College, which reviewed and approved this manuscript prior to submission.

## Abbreviations

AI/ANs: American Indians and Alaska Natives
aOR: Adjusted odds ratio
CI: Confidence interval
HIV: Human immunodeficiency virus
PWUID: People who use injection drugs
STI: Sexually transmitted infection
